# 4Ms Interventions in Hospitalized Older Adults: What are “Act-ons” for Positive Screens?

**DOI:** 10.1093/geroni/igaf122.3146

**Published:** 2025-12-31

**Authors:** Justin Lam, Susan Kwiatek, Shahidul Islam, Mark Tursi, Christian Nouryan, Philip Solomon, Maria Carney, Edith Burns

**Affiliations:** Donald and Barbara Zucker School of Medicine at Hofstra/Northwell, Brooklyn, New York, United States; Northwell Health, New Hyde Park, New York, United States; Northwell Health, New Hyde Park, New York, United States; Northwell Health, New Hyde Park, New York, United States; Northwell Health, Great Neck, New York, United States; Northwell Health, New Hyde Park, New York, United States; Zucker School of Medicine at Hofstra/Northwell, New Hyde Park, New York, United States; Zucker School of Medicine Hofstra-Northwell, Manhasset, New York, United States

## Abstract

The 4Ms framework -What Matters Most (WMM)/Medication/Mentation/Mobility- facilitates addressing issues critical to care of older adults. We identified different interventions, “Act-ons”, for positive screens in hospitalized patients assessed for all 4Ms. Unique patients identified by Age-Friendly Health System (AFHS) dashboard aggregating data on adults ≥65y/o admitted to one of 8 hospitals and assessed for all 4Ms. Individual EMR manually reviewed for demographic and clinical data, and for each positive M to determine Act-ons. Admissions reviewed from July-September 2024. A total of 297 patients received all 4Ms (9.6% of 3104 admissions). Median age: 84y/o (IQR:76-90); 57% female, (85%); race: 63% White, 13% Black/AA, 12% Asian, 13% other; 85% English speaking Median LOS: 9 days (IQR:6-15). All 297 had GOC documented but 58.3% did not include WMM. Act-ons: managing team discussed with patient (60%); palliative care consult (40%). For 121 positive Medication assessments, 57.8% had Act-ons: documented reason for no change (73.6%); decrease/deprescribed (26.4%). For 297 Mobility screens, 83.1% had Act-ons: 99% PT consult; 1% RN plan. For 13 positive Mentation screens, 2 (15.4%) had Act-on: electrolytes reviewed/reported; medication side effect treated. This is one of the first reports of Act-ons for 4Ms. Mobility had most Act-ons, then Medication, WMM, lastly Mentation. Act-ons were similar for 3 of the 4Ms across different hospitals within the health system. Delirium was essentially not detected across locations, suggesting need for a root-cause analysis and further education/training. In the future, structured processes for Act-ons may help ensure patient needs are optimally met.

